# Synergistic combinations of short high-voltage pulses and long low-voltage pulses enhance irreversible electroporation efficacy

**DOI:** 10.1038/s41598-017-15494-3

**Published:** 2017-11-09

**Authors:** Chenguo Yao, Yanpeng Lv, Yajun Zhao, Shoulong Dong, Hongmei Liu, Jianhao Ma

**Affiliations:** The State Key Laboratory of Power Transmission Equipment and System Security and New Technology, the School of Electrical Engineering, Chongqing University, Chongqing, 400030 China

## Abstract

Irreversible electroporation (IRE) uses ~100 μs pulsed electric fields to disrupt cell membranes for solid tumor ablation. Although IRE has achieved exciting preliminary clinical results, implementing IRE could be challenging because of volumetric limitations at the ablation region. Combining short high-voltage (SHV: 1600V, 2 μs, 1 Hz, 20 pulses) pulses with long low-voltage (LLV: 240–480 V, 100 μs, 1 Hz, 60–80 pulses) pulses induces a synergistic effect that enhances IRE efficacy. Here, cell cytotoxicity and tissue ablation were investigated. The results show that combining SHV pulses with LLV pulses induced SKOV3 cell death more effectively, and compared to either SHV pulses or LLV pulses applied alone, the combination significantly enhanced the ablation region. Particularly, prolonging the lag time (100 s) between SHV and LLV pulses further reduced cell viability and enhanced the ablation area. However, the sequence of SHV and LLV pulses was important, and the LLV + SHV combination was not as effective as the SHV + LLV combination. We offer a hypothesis to explain the synergistic effect behind enhanced cell cytotoxicity and enlarged ablation area. This work shows that combining SHV pulses with LLV pulses could be used as a focal therapy and merits investigation in larger pre-clinical models and microscopic mechanisms.

## Introduction

Electroporation has been widely used to increase cell membrane permeabilization through pores by employing a high-voltage pulsed electric field^1^. After the electric pulse, pores in the cell membrane may persist for a few seconds to a few minutes with cell survival, which is known as reversible electroporation^2–5^. This process can be used to achieve intracellular uptake of various molecules^6–8^, electrofusion^9,10^, and nanoelectroporation^11,12^. When stronger electric pulses act on the cell membrane, the pore may become too large to recover, which causes irreversible damage to the cell membrane and thus leads to cell death. However, electroconformational protein denaturation, osmotic imbalance, and a flush in/out of ions could occur before complete pore reseal and may result in cell death. Moreover, cells may also die by apoptosis due to change in homeostasis. This phenomenon is called irreversible electroporation (IRE)^13,14^, which is used for bacterial inactivation^15^, tumor ablation^16,17^, and food processing^18^.

IRE was recently developed as a new minimally invasive and non-thermal ablation technology for tumor treatment^19–21^. The typical IRE treatment protocol delivers tens of electric pulses with a duration of ~100 μs at hundreds to thousands of V/cm using two or more needle electrodes^22,23^. The targeted tumor ablation area is controlled by adjusting the electrode arrangement and the pulse parameters^23,24^. Particularly, one review^25^ recently analyzed the safety and efficacy of IRE treatments in 16 clinical studies including 221 patients with advanced malignancies of the liver, pancreas, kidneys, lesser pelvis, lungs and lymph nodes. The studies found that IRE is safe and efficient for application in small human tumors including those located around blood vessels and bile ducts. Savic *et al*.^26^ also reviewed IRE in a clinical application and provided a state-of-the-art update on the available clinical evidence of IRE regarding its feasibility, safety and oncologic efficacy. However, IRE seems to have disappointing and less promising results with respect to lung cancer.

Although IRE has achieved exciting clinical results, treatments are restricted to relatively small tumors of less than 3 cm and decrease with increasing the sizes of tumors^27–30^. Increasing the pulse parameters (applied pulse voltage, pulse width, pulse number, etc.) during IRE can increase the affected area. However, increasing the pulsed electrical power delivered may result in thermal effects, which IRE attempts to avoid^31–33^. On the other hand, increasing the affected area can also be achieved by using multiple electrodes. However, this adds to the complexity of the procedure and increases the operative time due to the arrangement of multiple electrodes^25,34^. Therefore, some researchers have focused on enlarging the ablation area by employing electric pulses with two needle electrodes. Rubinsky *et al*.^35,36^ showed that combining electroporation by electric pulses with electrolysis by DC current yielded larger tissue ablation compared to electroporation or electrolysis delivered separately. Muratori *et al*.^37^ found that the ablation area could be increased by splitting a high-rate trains of nanosecond pulses into two identical trains with a sufficiently long interval. In addition, Jiang *et al*.^38^ found that splitting the trains of IRE pulses into several trains enhances cell death. Ivey *et al*.^39^ found that high frequency IRE has more size-selective lethal thresholds compared to traditional IRE when ablating glioblastoma multiforme tumors. Moreover, Sano *et al*.^40^ found that asymmetric high frequency IRE produced a larger ablation zone compared to symmetric high frequency IRE. On the other hand, Frandsen *et al*.^41^ also found that calcium electroporation could increase ablated zone compared to calcium alone.

A method for tissue ablation was recently proposed based on pulsed electric fields by combining short high-voltage (SHV) pulses with long low-voltage (LLV) pulses to enhance the ablation area. This combination may induce a synergetic effect and significantly enhance the tissue ablation area. SHV pulses created a large electroporated area that was more susceptible to subsequent LLV pulses, therefore significantly enhancing the ablation area. This modality of pulsed electric fields was reported in a previous study to enlarge the ablation area of potato tissue *in vitro* ^42^. However, enhancement of the cell cytotoxic effect by combining SHV pulses with LLV pulses has not yet been explored. Furthermore, the ability of this modality of pulsed electric fields to enlarge the ablation area in animals *in vivo* has not been established.

In this study, the response of tumor cells in a media suspension was used as a surrogate to investigate the enhancement of the cell cytotoxic effect by combining SHV pulses (2 μs) with LLV (100 μs) pulses. The results showed that cell viability was below 20% by combining SHV pulses with LLV pulses, while cell viability was maintained above 60% when either SHV pulses or LLV pulses were applied alone. Furthermore, when the lag time between the SHV pulse and LLV pulse protocols was adjusted to 100 s, cell viability further decreased (below 10%). The liver tissues of New Zealand white rabbits were selected as an *in vivo* research model to study the ablation zone following application of the electric pulses. The results also showed that the liver ablation area was enlarged when the synergetic effect was evoked by combining SHV pulses (2 μs) with LLV (100 μs) pulses compared to when either SHV pulses or LLV pulses were applied alone. Moreover, prolonging the lag time between the SHV pulse and the LLV pulse protocols also further enlarged the ablation area. However, the order in which the SHV and LLV pulses were applied was important, and only the SHV + LLV pulse sequence increased both the cell cytotoxic effect and ablation area. The pulsed electric fields that are used may be applied to optimize the current pulsed electric protocols and to enhance the outcome of IRE efficacies.

## Materials and Methods

### Cytotoxicity in cells exposed in cuvettes

Human ovarian carcinoma cells (SKOV-3) were donated by the Basic Medical Science College, Chongqing Medical University, Chongqing, China. The cells were washed with 1–2 mL phosphate-buffered saline and were digested with 0.5 mg/mL trypsin. After 4 min, the trypsin was removed, and 1640 culture media was added. A cell suspension with a density of 5.0 × 10^5^ cells/mL was obtained, and 100 μL cell suspension was injected into a 4-mm gap cuvette (Harvard Apparatus, Holliston, MA). The pulse generator that was used in this study was developed in the laboratory. The samples remained in cuvettes until the exposure experiment was completed. The samples that included the different experimental parameter groups and non-exposed sham control group were aliquoted into a 96-well plate in quintuplicates of 10 × 10^3^ cells/well and incubated at 37 °C. The next day (22–24 h after exposure), 20 μL CCK-8 (Beyotime Co., Ltd., Jiangshu, China) was added to each well, and the cells were incubated for another 1 h at 37 °C. The absorbance was then measured at 450 nm with an enzyme-linked immunometric meter (BIO-RAD, CA). The quintuplicate data were averaged without the maximal and minimal data and considered to be a single experiment. The percentage of cell viability was determined as

1 $$Viability=\frac{{I}_{sample}-{I}_{media}}{{I}_{conrtol}-{I}_{media}}\times 100 \% ,$$

where I_sample_ is the relative intensity measurement of experimental group from the spectrophotometer, I_control_ is the relative intensity measurement of non-exposed sham control group from the spectrophotometer, I_media_ is the relative intensity measurement of culture media without cells from the spectrophotometer.

### Experimental animals

The experiments using animals included 15 New Zealand rabbits (female, 6 months old, 2.5 ± 0.2 kg weight), which were obtained from the Experimental Animal Center of Chongqing Medical University. The rabbits were housed in individually ventilated cages with access to food and water ad libitum and were maintained in a temperature-controlled room. All protocols were approved by the Ethics Committee of Chongqing Medical University, and the experiments were performed in accordance with the guidelines.

### Experimental procedures for liver ablation *in vivo*

**Figure 1:**
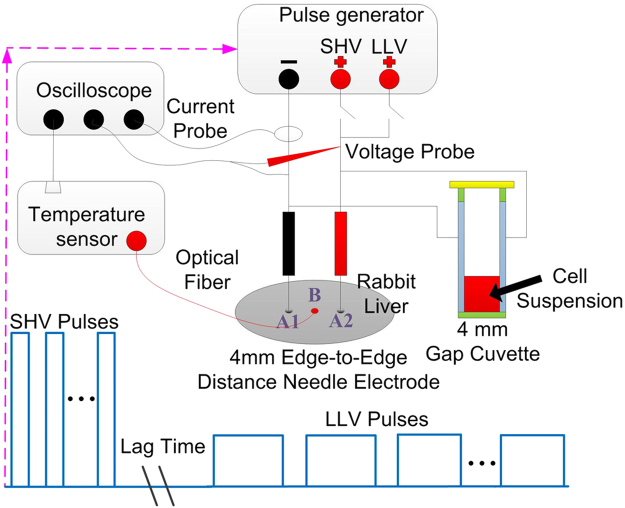
Experimental setup and illustration of the cell ablation and liver ablation experiments. The electrodes were 1 mm in external diameter, 4 mm in edge-to-edge distance and 8 mm in exposed length.

**Figure 2:**
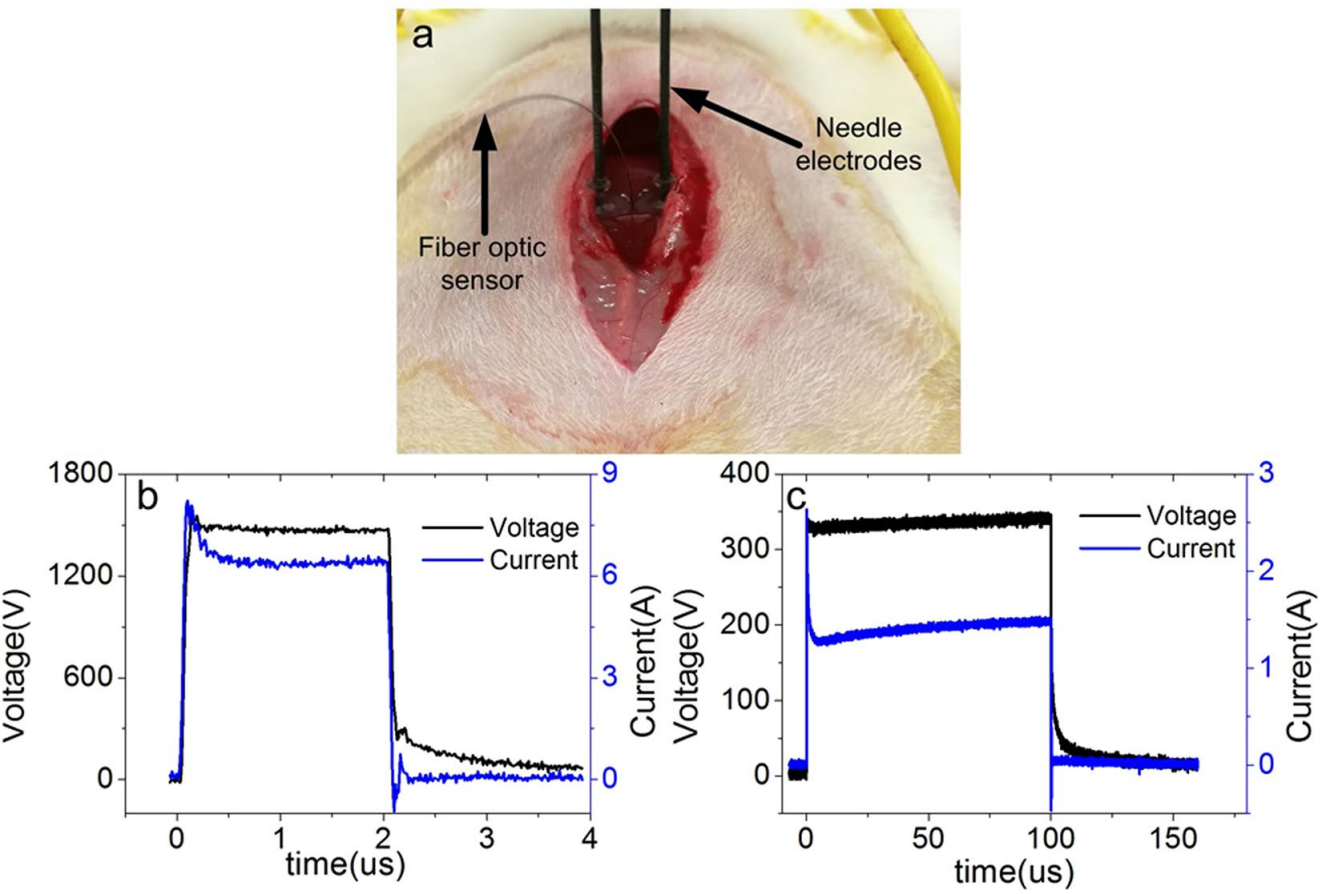
Rabbit liver ablation experiments. (**a**) Digital photograph of the experiment. The fiber optic sensor is inserted between the two needle electrodes. Typical voltage and current waveforms with (**b**) a SHV pulse (1,600 V, 2 μs) and (**c**) a LLV pulse (360 V, 100 μs).

Rabbits were anesthetized with an injection of 2.5 mL 3% sodium pentobarbital solution to the ear edge vein ten minutes before the experiments. Anesthesia was induced for approximately 2 h, which was sufficient for the experiments. The animal was then placed in a supine position on a sterile surgical table. Muscle relaxants were not administered to the rabbits. The rabbits’ legs were tied down to aid in the operation. The abdomen was opened with a 50-mm midline incision so that the pulses could be applied to different lobes (Fig. 1). A picture of the electrode positions on the liver of a rabbit during the pulse delivery is shown in Fig. 2(a). The electrodes were made from medical stainless steel, which has been used in clinical applications of IRE. The electrodes were 1 mm in external diameter, 4 mm in edge-to-edge distance and 8 mm in exposed length. Real-time temperature was recorded using a fiber optic Luxtron m600 OEM sensor (FOT Lab Kit, LumaSense Technologies, Santa Clara, USA). The temperature measurement probes were inserted between the needle electrodes during the experiments. Rabbits have three big lobes (except one lobe containing the gall bladder). Ablation was attempted on each lobe. After the treatment, minor bleeding was observed, and sterile gauze was used to stop the bleeding. The edges of the wound were stitched together with silk sutures. No abnormalities were observed during the entire ablation procedure.

### Histology processing

The tissue specimens were harvested 72 h following the ablation experiments. Liver samples were fixed in 10% formalin, embedded in paraffin, and sectioned and processed for histology using hematoxylin and eosin (H&E) staining. Color images of each tissue section were acquired using an Aperio LV1 Digital Pathology Slide Scanner (Leica Biosystems Inc., Buffalo Grove, USA). Pathologists of the Third Military Medical University who were not involved in the experiment evaluated the periphery of the ablation area in both fresh liver tissue samples and the H&E images. ImageJ software was used to calculate the ablation area in the photographs of fresh liver tissue samples.

### Pulse generator, parameter and electrodes

The cell ablation and liver ablation equipment that was used in this study was developed in the laboratory and comprised a pulse generator with dual power charging through the MOSFET circuit to produce combined SHV pulses with LLV pulses and its supporting electrodes for liver ablation (4-mm gap cuvette for cell suspension). The experimental setup and illustrations of the cell ablation and liver ablation experiments are shown in Fig. 1. The output voltages and currents were measured *in vivo* (in Fig. 1) using a WavePro 760Zi-A oscilloscope (Teledyne LeCroy Inc., New York, USA) with a PPE-5 kV high voltage probe and a Pearson current probe 6600 (Pearson Electronics Inc., Palo Alto, USA). The voltage and current waveforms recorded during experimentation are shown in Fig. 2(b) and (c). For the experiment of cell cytotoxicity and rabbit liver ablation, the parameter of SHV pulses used 1,600 V, 2 μs, and 10–80 pulses, and the parameter of LLV pulses used 240–480 V, 100 μs, and 60–80 pulses. In the SHV + LLV pulse protocol, LLV pulses were applied after SHV pulses, with a lag time of 1 s and 100 s, while in the LLV + SHV pulse protocol, the sequence was reversed. The exposure parameters that correspond to each figure were listed in the results section. The electrical dose was used to facilitate a comparison, as described by the following equation^43^:

2 $$Dose=\sum _{n=1}^{N}{V}_{n}^{2}\times {T}_{n}[{V}^{2}s]$$

where *V*_n_ is the voltage of the *n*th pulse, *T*_n_ is the width of the *n*th pulse, and *N* is the total number of pulses.

The finite element models of the electric field distribution and temporal temperature solution were built to obtain the electric field thresholds to correlate with the liver ablation zones. The detailed simulations can be found in the Supplemental material. The electric field distribution with contours was calculated, and the curve between the electric field and the area was then drawn. The electric field where the area was equal to the ablation area in the fresh liver sample was the lethal electric field threshold.

### Statistical analysis

All data were statistically analyzed by performing ANOVA using Microsoft Excel. The data are presented as the means ± standard deviation (SD), and the significance of the indexes between the different parameter groups was tested. All data are fully available without restriction.

## Results

### Cell cytotoxic enhancement by combining SHV pulses with LLV pulses

**Figure 3:**
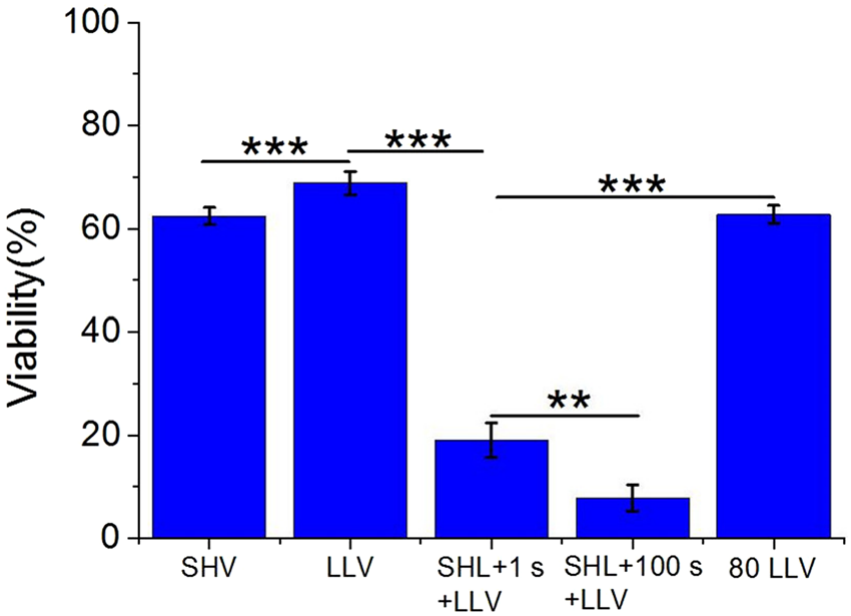
Cell viability resulting from SHV pulses, LLV pulses, SHV pulses + 1 s + LLV pulses and SHV pulses + 100 s + LLV pulses (the parameters were given in Table 1). The cell viability after SHV + LLV pulses was lower than that after either SHV or LLV pulses applied alone. Prolonging the lag time (100 s) also enhanced cell cytotoxicity. **p < 0.01, ***p < 0.001.

In Fig. 3, cell viability is presented where SHV pulses in combination with LLV pulses, SHV pulses and LLV pulses were applied, respectively (in Table 1). The cell viability was above 60% when either 10 SHV pulses (62 ± 2%) or 60 LLV pulses (69 ± 2%) were applied alone, which contributed less to SKOV-3 cell cytotoxicity. However, the cell viability was only 19 ± 3% when 10 SHV pulses and 60 LLV pulses were combined. The cell viability after LLV pulses was 3.61 times that after SHV + LLV pulses. The cell viability measured after SHV pulses was 3.28 times that after SHV pulses and LLV pulses combined. There was a significant difference between the combination of SHV pulses with LLV pulses and SHV pulses (p < 0.001) applied alone or LLV pulses (p < 0.001) applied alone. Moreover, when 80 LLV pulses were applied, the cell viability was 63 ± 2%, which was a little lower than that after 60 LLV pulses (69 ± 2%) but significantly higher than that (19 ± 3%) after 10 SHV pulses with 60 LLV pulses (p < 0.001). The above experiment was performed to verify that the evoked synergistic effect of combining SHV pulses with LLV pulses increased cell cytotoxicity. In addition, cell viability was further decreased (8 ± 3%) when the lag time between SHV pulses and LLV pulses reached 100 s. There was also a significant difference (p < 0.01) in cell viability observed for different lag times (1 s and 100 s).

**Table 1:** Cell experimental parameters for the setups shown in Fig. 3, n = 3.

Parameters	SHV pulses			Lag time	LLV pulses			Sequence	Cell viability (%)
	Voltage (V)	Width (μs)	Number		Voltage (V)	Width (μs)	Number		
Fig. 3	1,600	2	10					SHV	62 ± 2
					240	100	60	LLV	69 ± 2
	1,600	2	10	1 s	240	100	60	SHV + LLV	19 ± 3
	1,600	2	10	100 s	240	100	60	SHV + LLV	8 ± 3
					240	100	80	LLV	63 ± 2

**Figure 4:**
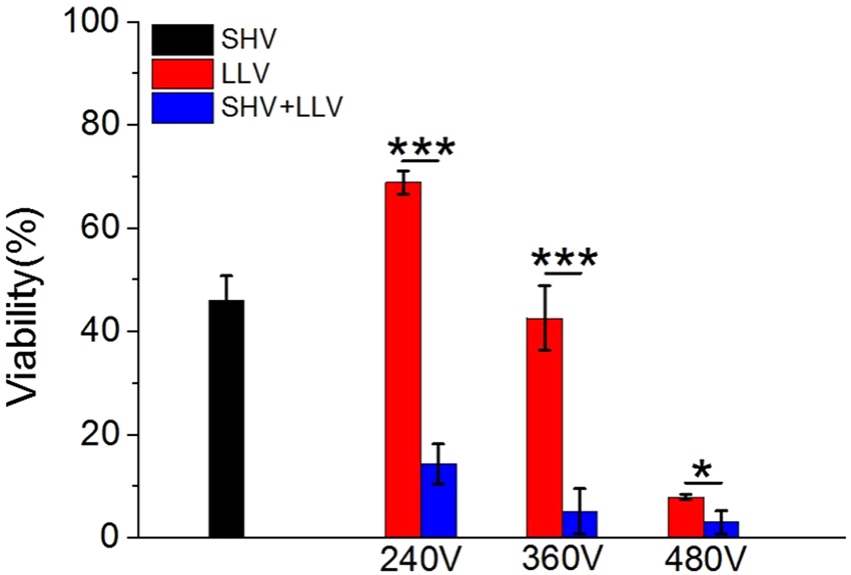
Cell viability resulting from SHV + LLV pulses was lower than that after either SHV or LLV pulses applied alone on the condition that the same voltage of LLV pulses in the range of 240–480 V was applied (the parameters were given in Table 2). *p < 0.05, **p < 0.01, ***p < 0.001.

Synergistic effects still existed when combining the 20 SHV pulses with 60 LLV pulses were applied. As shown in Fig. 4 (the pulse parameters were given in Table 2), the cell viability (14 ± 4%) observed after combining 20 SHV pulses with 60 LLV (240 V) pulses was less than that observed for 20 SHV pulses applied alone (46 ± 5%) and 60 LLV (240 V) pulses applied alone (69 ± 2%). Moreover, the cell viability was decreased after the electric field of LLV pulses applied alone was increased from 240 V to 480 V. The cells pretreated with 20 SHV pulses amplified this trend. For LLV (360 V) pulses applied alone, the cell viability was 43 ± 6%. For SHV pulses applied alone, the cell viability was 46 ± 5%, which was similar to the viability that resulted from LLV (360 V) pulses applied alone. However, cell death was significantly enhanced (cell viability: 5 ± 4%) when SHV pulses were combined with LLV (360 V) pulses. The cell viability resulting from LLV (360 V) pulses applied alone was 8.25 times that after SHV pulses combined with LLV (360 V) pulses. The cell viability that resulted from SHV pulses applied alone was 8.91 times that after SHV pulses combined with LLV (360 V) pulses. There was a significant difference between the 20 SHV + 60 LLV (360 V) combination and 20 SHV pulses alone (p < 0.001) or 60 LLV (360 V) pulses alone (p < 0.001). When the voltage of LLV pulses increased to 480 V, the cell viability was 8 ± 1% when LLV (480 V) pulses were applied alone. However, cell viability decreased to 3 ± 2% when SHV pulses were combined with LLV (480 V) pulses. There was also a significant difference (p < 0.05) between the SHV + (480 V) LLV pulses and LLV (480 V) pulses.

**Table 2:** Cell experimental parameters for the setups shown in Fig. 4, n = 3.

Parameters	SHV pulses			Lag time	LLV pulses			Sequence	Cell viability (%)
	Voltage (V)	Width (μs)	Number		Voltage (V)	Width (μs)	Number		
Fig. 4	1,600	2	20					SHV	46 ± 5
					240	100	60	LLV	69 ± 2
	1,600	2	20	1 s	240	100	60	SHV + LLV	14 ± 4
					360	100	60	LLV	43 ± 6
	1,600	2	20	1 s	360	100	60	SHV + LLV	5 ± 4
					480	100	60	LLV	8 ± 1
	1,600	2	20	1 s	480	100	60	SHV + LLV	3 ± 2

**Figure 5:**
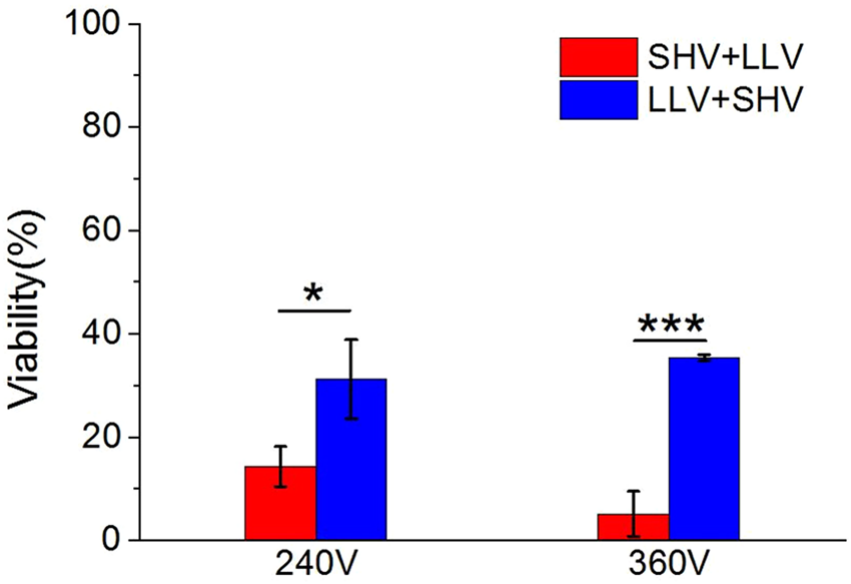
Sequence of the application of SHV pulses and LLV pulses influenced cell viability (the parameters were given in Table 3). LLV + SHV pulses were not as effective as SHV + LLV pulses. *p < 0.05, ***p < 0.001.

SHV + LLV pulses had a positive effect on cell cytotoxicity. However, the LLV + SHV combination was not as effective as SHV + LLV. Figure 5 showed the cell viability observed for two pulse protocols (as shown in Table 3), i.e., SHV + LLV pulses and LLV + SHV pulses. The SHV + LLV pulses induced greater cell death than the LLV + SHV pulses. There was also a significant difference between the two sequences. Therefore, the sequence may play an important role in cell death via a synergistic effect of combining the SHV pulses with LLV pulses.

**Table 3:** Cell experimental parameters for the setups shown in Fig. 5, n = 3.

Parameters	SHV pulses			Lag time	LLV pulses			Sequence	Cell viability (%)
	Voltage (V)	Width (μs)	Number		Voltage (V)	Width (μs)	Number		
Fig. 5	1,600	2	20	1 s	240	100	60	SHV + LLV	14 ± 4
								LLV + SHV	31 ± 8
	1,600	2	20	1 s	360	100	60	SHV + LLV	5 ± 4
								LLV + SHV	35 ± 1

**Figure 6:**
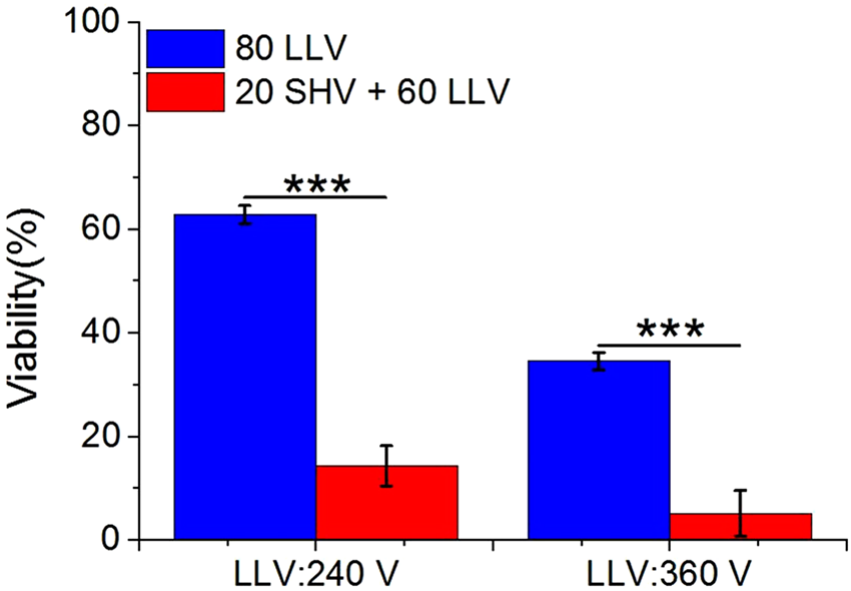
Cell viability resulting after 80 LLV pulses was higher than that after 20 SHV pulses + 60 LLV pulses (the parameters were given in Table 4). **p < 0.01, ***p < 0.001.

When 80 LLV pulses were applied alone (keeping the same pulse number with 20 SHV + 60 LLV pulses), the synergistic effect still existed. As shown in Fig. 6, the cell viability (63 ± 2%) after 80 LLV (240 V) pulses was also lower than that (14 ± 4%) after combining 20 SHV pulses with 60 LLV (240 V) pulses when keeping the same pulse number with 20 SHV + 60 LLV pulses. When the voltage of LLV pulses was increased to 360 V, the cell viability after 80 LLV (360 V) pulses was 35 ± 2%, which was still higher than that (5 ± 4%) after combining 20 SHV pulses with 60 LLV (360 V) pulses. Therefore, the cell viability when of 20 SHV pulses were applied before 60 LLV pulses was still lower than that after 80 LLV pulses.

**Table 4:** Cell experimental parameters for the setups shown in Fig. 6, n = 3.

Parameters	SHV pulses			Lag time	LLV pulses			Sequence	Cell viability (%)
	Voltage (V)	Width (μs)	Number		Voltage (V)	Width (μs)	Number		
Fig. 6					240	100	80	LLV	63 ± 2
	1,600	2	20	1 s	240	100	60	SHV + LLV	14 ± 4
					360	100	80	LLV	35 ± 2
	1,600	2	20	1 s	360	100	60	SHV + LLV	5 ± 4

**Figure 7:**
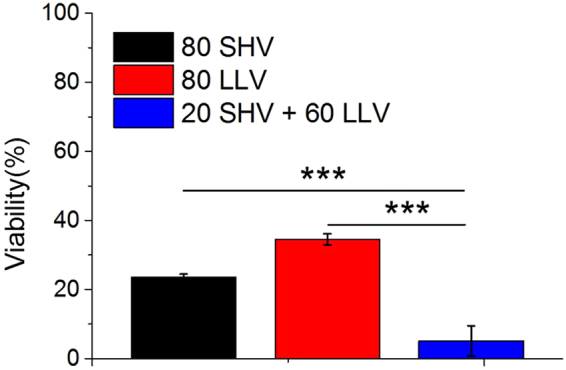
Cell viability that resulted from 80 SHV pulses, 80 LLV pulses and 20 SHV pulses + 60 LLV pulses (the parameters were given in Table 5). 20 SHV + 60 LLV pulses were still highly effective when keeping the same number with either 80 SHV pulses or 80 LLV pulses. ***p < 0.001.

To further investigate the high efficiency of SHV + LLV pulses for cell killing under the same pulse number, the application of 80 SHV (1600 V) pulses or 80 LLV (360 V) pulses was used to maintain the same pulse number with 20 SHV + 60 LLV pulses. The cell cytotoxicity after combining 20 SHV pulses with 60 LLV pulses was still larger than that after either 80 SHV pulses or 80 LLV pulses applied. As shown in Fig. 7, when 80 SHV pulses were applied, the cell viability was 24 ± 1%, which was lower than that (46 ± 5%) after 20 SHV pulses (See Fig. 4). When 80 LLV (360 V) pulses were applied, the cell viability was 35 ± 2%, which was also lower than that (43 ± 6%) after 60 LLV (360 V) pulses (See Fig. 4). However, the cell viability that resulted from 20 SHV + 60 LLV (360 V) pulses was 5 ± 4%, which was significantly lower than that after 80 LLV pulses (p < 0.001) or 80 SHV pulses (p < 0.001). Therefore, on the condition that the 20 SHV + 60 LLV pulses have the same pulse number as 80 SHV pulses or 80 LLV pulses, applying either 80 SHV pulses or 80 LLV pulses was still less effective than that by 20 SHV + 60 LLV pulses.

**Table 5:** Cell experimental parameters for the setups shown in Fig. 7, n = 3.

Parameters	SHV pulses			Lag time	LLV pulses			Sequence	Cell viability (%)
	Voltage (V)	Width (μs)	Number		Voltage (V)	Width (μs)	Number		
Fig. 7	1,600	2	80					SHV	24 ± 1
					360	100	80	LLV	35 ± 2
	1,600	2	20	1 s	360	100	60	SHV + LLV	5 ± 4

**Figure 8:**
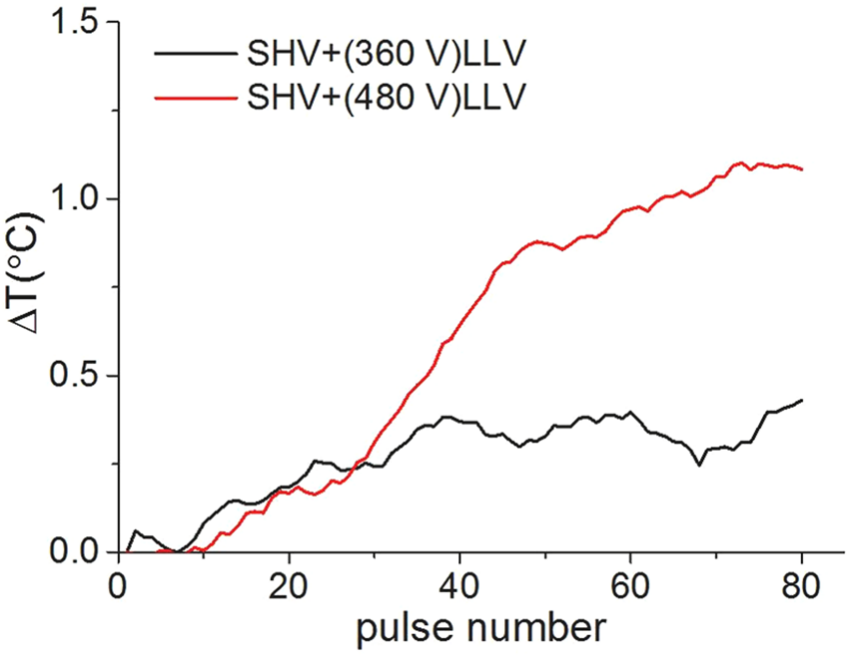
Mean temperature rise that resulted from combining SHV pulses with LLV pulses in cell suspension. Parameters of the black line: 20 SHV (1,600 V, 2 μs) pulses + 60 LLV (360 V, 100 μs) pulses. Parameters of the red line: 20 SHV (1,600 V, 2 μs) pulses + 60 LLV (480 V, 100 μs) pulses.

Temperature rise is an important consideration when combining SHV pulses with LLV pulses and should be kept within given limits. As shown in Fig. 8, the temperature rise data are presented as the mean value of three independent experiments. The maximum temperature rise was 0.43 °C upon combining SHV pulses with LLV (360 V) pulses. The maximum temperature increase was 1.08 °C for the combined SHV pulses with LLV (480 V) pulses. Therefore, a small temperature rise may occur when combining SHV pulses with LLV pulses.

### Ablation area enlargement by combining SHV pulses with LLV pulses

**Figure 9:**
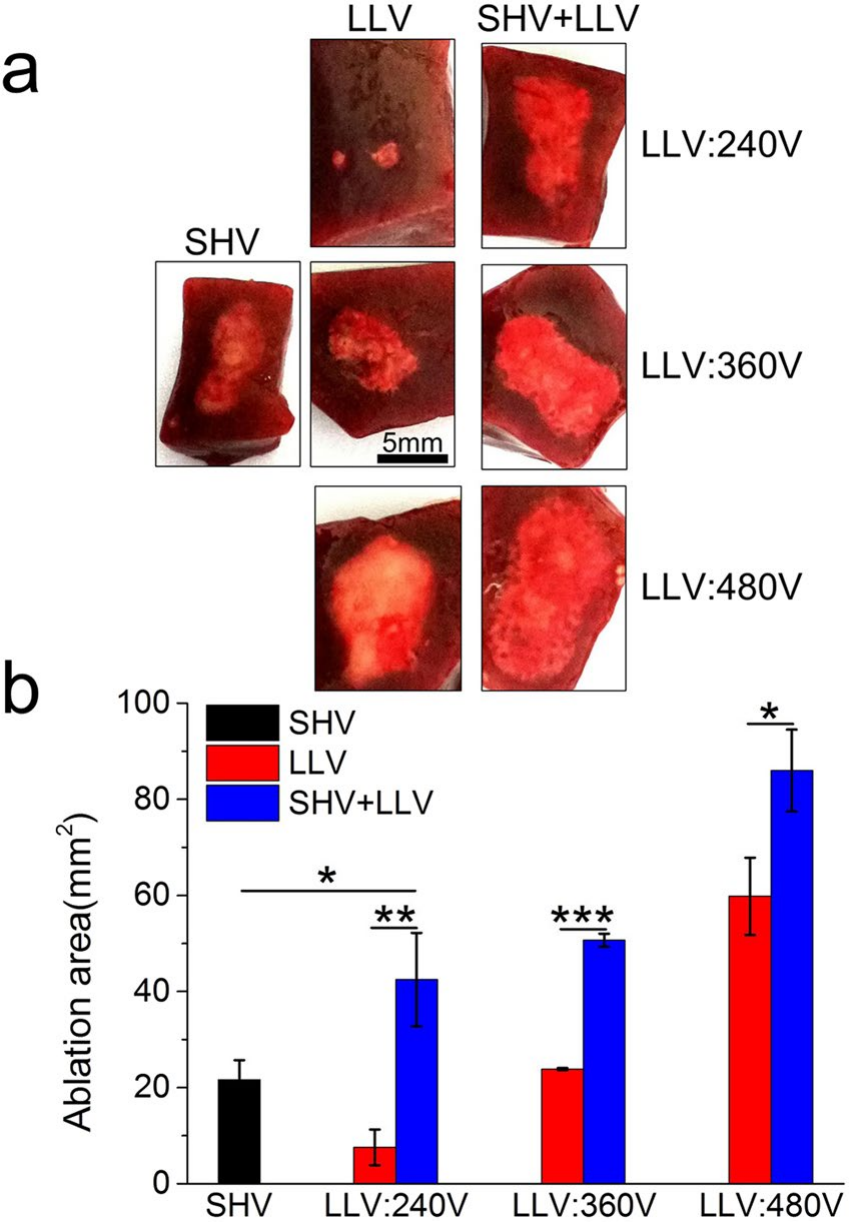
SHV + LLV pulses ablated tissues more effectively compared to either SHV or LLV pulses applied alone (the parameters were given in Table 6). (**a**) Ablation areas of fresh liver samples and (**b**) Mean ablation areas induced from SHV pulses, LLV pulses, and SHV + LLV pulses. *p < 0.05, **p < 0.01, ***p < 0.001.

**Figure 10:**
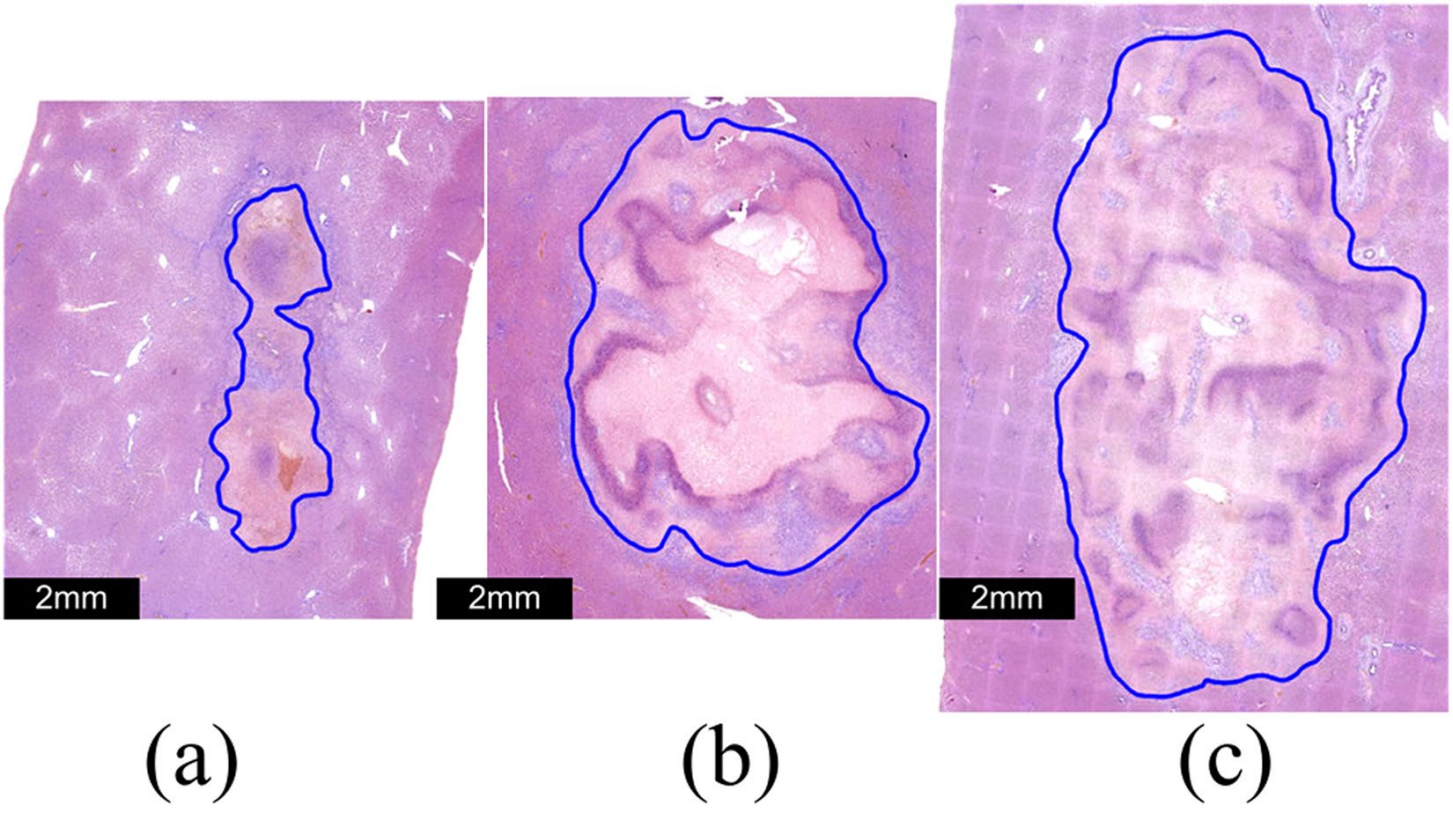
Comparison of tissue sections with H&E staining after application of (**a**) SHV pulses (left), (**b**) LLV (480 V) pulses (middle) and (**c**) a combination of SHV pulses with LLV (480 V) pulses (right).

As shown in Fig. 9 (the pulse parameters are given in Table 6), lesions were clearly visible as ellipsoidal hemorrhagic regions that were centered between the electrodes in the fresh liver samples. The bright red area indicates the region of cell death. The ablation area was approximately 21.65 mm^2^ after SHV pulses were applied alone. The ablation area was increased after the voltage of LLV pulses applied alone was increased from 240 V to 480 V. However, liver tissues pretreated with SHV pulses (SHV + LLV) amplified this trend. As shown in Fig. 9, applying LLV (240 V) pulses produced a very small ablation area (7.58 mm^2^) that evolved from two separate circles around the electrodes. However, when LLV (240 V) pulses were applied after SHV pulses, the ablation area was significantly enhanced to approximately 42.47 mm^2^, which was 460.29% larger than that after LLV (240 V) pulses applied alone. However, the dose of combining SHV pulses and LLV (240 V) pulses was 448 V^2^s, which was 29.63% larger than the dose of LLV (240 V) pulses applied alone. When the voltage of LLV pulses was adjusted to 360 V and applied alone, the ablation area increased to 23.85 mm^2^. Although the dose (880 V^2^s) of combining SHV pulses and LLV (360 V) pulses was only 13.17% larger than the dose of LLV (360 V) pulses applied alone, combining SHV pulses with LLV (360 V) pulses yielded an ablation area of 50.70 mm^2^, which was increased by 112.58% relative to that after LLV (360 V) pulses applied alone. Even for 480 V LLV pulses, the dose (1,484.8 V^2^s) of combining SHV pulses and LLV (480 V) pulses was only 7.41% larger than the dose of LLV (480 V) pulses applied alone, but the ablation area was 43.76% larger than that after LLV (480 V) pulses applied alone (59.82 mm^2^). After a fixation in 10% formalin and H&E staining, the image analysis revealed that, although the ablation area was shrunk, combining SHV pulses with LLV (480 V) pulses still induced a markedly larger ablation area than the ablation area from either SHV pulses or LLV (480 V) pulses applied alone (Fig. 10). Therefore, by combining SHV pulses with LLV pulses, a synergetic effect was also evoked that enhanced the liver ablation area.

**Table 6:** Liver experimental parameters for the setups shown in Fig. 9, n = 3.

Parameters	SHV pulses			Lag time	LLV pulses			Sequence	Dose (V^2^s)	Ablation area (mm^2^)
	Voltage (V)	Width (μs)	Number		Voltage (V)	Width (μs)	Number			
Fig. 9	1,600	2	20					SHV	102.4	21.65 ± 4.06
					240	100	60	LLV	345.6	7.58 ± 3.71
	1,600	2	20	1 s	240	100	60	SHV + LLV	448	42.47 ± 9.74
					360	100	60	LLV	777.6	23.85 ± 0.22
	1,600	2	20	1 s	360	100	60	SHV + LLV	880	50.70 ± 1.34
					480	100	60	LLV	1,382.4	59.82 ± 8.04
	1,600	2	20	1 s	480	100	60	SHV + LLV	1,484.8	86.00 ± 8.51

**Figure 11:**
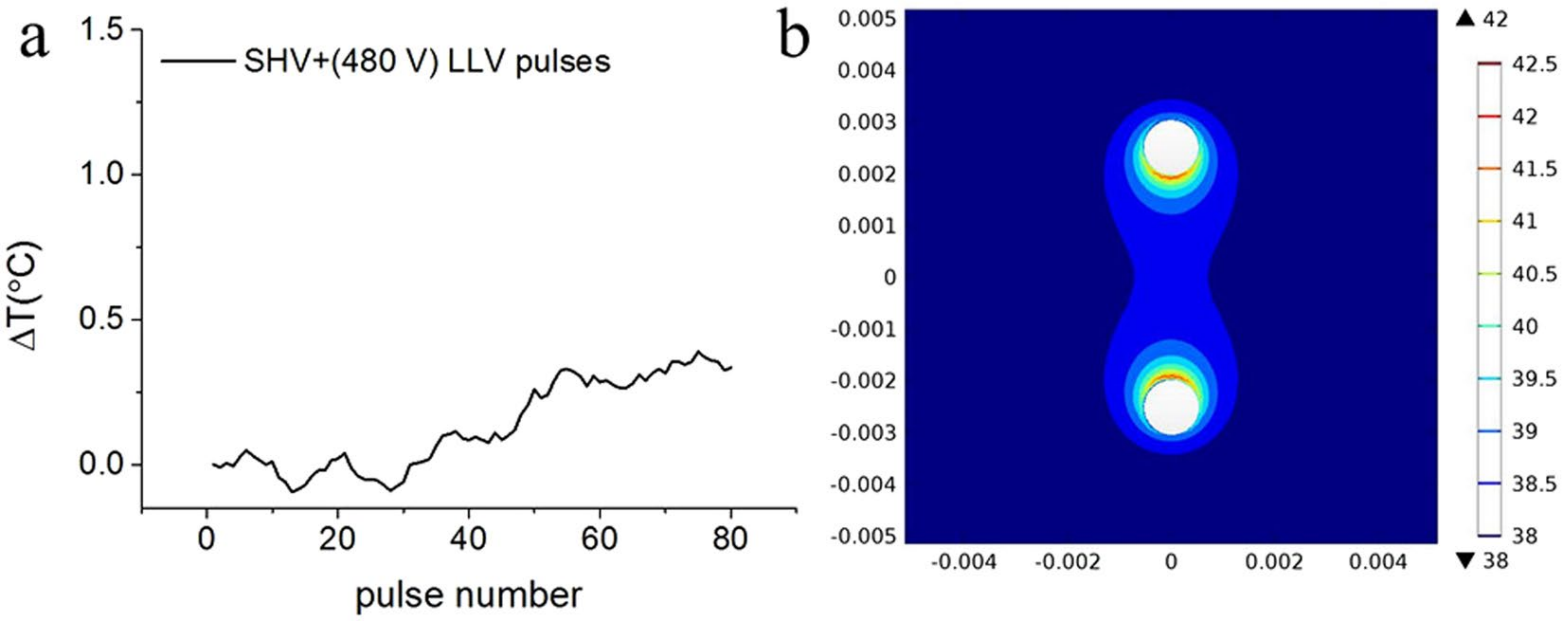
Measured temperature rise at the middle of the two needle electrodes (**a**) and temperature distribution in the simulation (**b**) resulting from the combination of 20 SHV pulses with 60 LLV (480 V) pulses during liver ablation; the measured temperature rise data are presented as the mean value of three independent experiments.

As shown in Fig. 11, the temperature increase after combining 20 SHV pulses and 60 LLV (480 V) pulses was 0.33 °C at the middle of the two needle electrodes (see Fig. 11a). Moreover, the simulation of 20 SHV pulses + 60 LLV (480 V) pulses predicted a temperature increase of 0.58 °C at the middle of the two needle electrodes. This simulation used a modified duty cycle approach, thus the predicted temperature increase was higher than the measured temperature increase because of less time for cooling between pulses in the simulation. Davalos *et al*.^44^ also found that the simulation results of temperature increase were higher compared to experimental data. In addition, they evaluated the thermal considerations relevant when applying IRE, then thought that clinical IRE therapy generated thermal effects, which might moderate the non-thermal aspects of IRE ablation. Therefore, clinical applications of IRE should consider thermal effects and employ protocols to ensure safe and effective therapy delivery. On the other hand, Faroja *et al*.^45^ found that whenever temperatures greater than 60 °C were seen, a thick rim of Hsp70, a marker of thermal damage, was noted peripheral to the zone of ablation. In this simulation, the maximum temperature was 42 °C near the electrode tips (see Fig. 11b) which was less than 60 °C, but biological assays, such as immunohistochemistry, should be further conducted to evaluate the thermal effects of the SHV + LLV combination.

**Figure 12:**
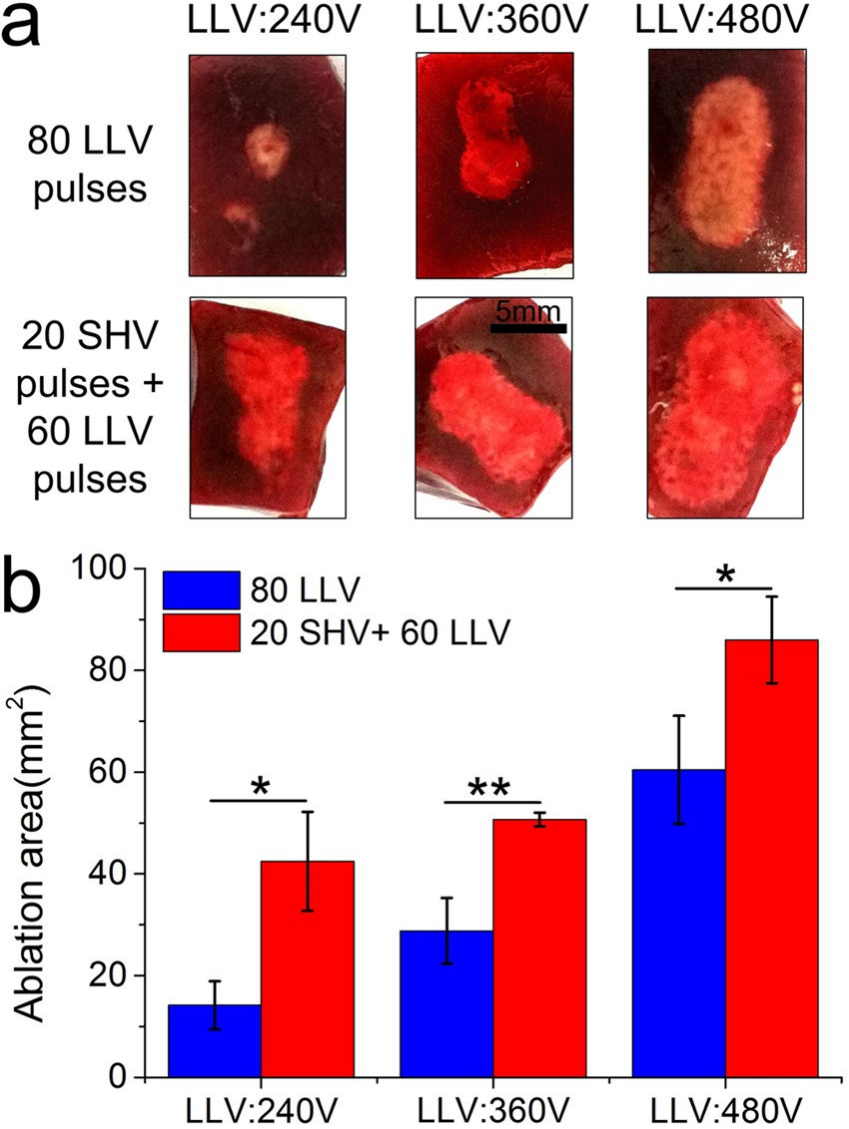
Applying the combination of 20 SHV pulses and 60 LLV pulses generated a larger ablation region with a lower dose than applying 80 LLV pulses. (the parameters were given in Table 7). (**a**) Ablation areas of fresh liver samples and (**b**) Mean ablation areas induced from 80 LLV pulses and 20 SHV pulses + 60 LLV pulses. *p < 0.05, **p < 0.01.

**Figure 13:**
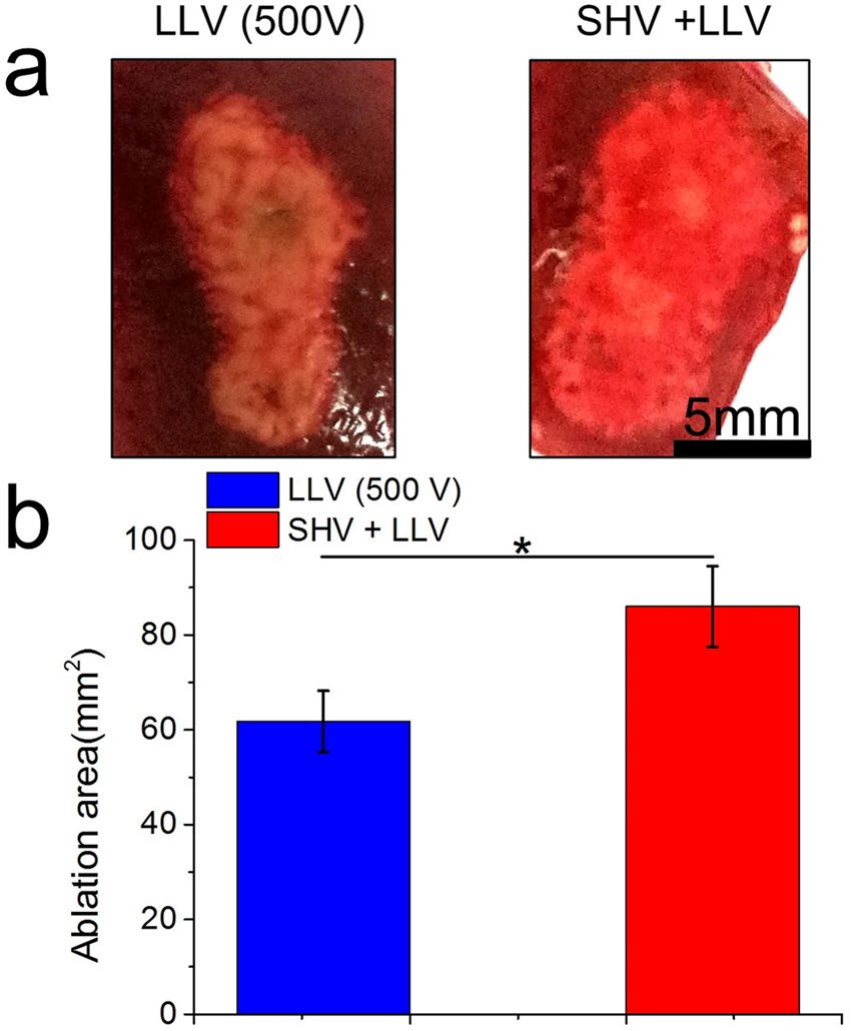
Ablation area after SHV + LLV (480 V) pulses was larger than that after 500 V (1,000 V/cm voltage-to-distance ratio), 100 μs, and 90 pulses as clinical IRE protocols (the parameters were given in Table 8). (**a**) Ablation areas of fresh liver samples and (**b**) Mean ablation areas induced from 500 V LLV pulses as well as 20 SHV pulses + 60 LLV (480 V) pulses. *p < 0.05.

When 80 LLV pulses were applied alone (keeping the same pulse number with 20 SHV + 60 LLV pulses), although the dose of combining 20 SHV pulses and 60 LLV pulses was lower than the dose of 80 LLV pulses, the synergistic effect still existed. As shown in Fig. 12, the dose (448 V^2^s) of combining 20 SHV pulses and 60 LLV (240 V) pulses was slightly lower than the dose (460.8 V^2^s) of 80 LLV (240 V) pulses. However, the ablation area (42.47 mm^2^) was still 199.08% larger than that (14.20 mm^2^) after 80 LLV (240 V) pulses. Moreover, for the 360 V and 480 V of LLV pulses, the ablation area from SHV + (360 V and 480 V) LLV pulses was still 75.92% and 42.27% larger than that by 80 LLV pulses with 360 V and 480 V respectively. However, the dose from SHV + (360 V and 480 V) LLV pulses was 17.82% and 24.12% lower than that by 80 LLV pulses with 360 V and 480 V respectively. Therefore, applying the combination of 20 SHV pulses and 60 LLV pulses also generated a larger ablation region with a lower dose than that by 80 LLV pulses. In addition, this study also used 500 V (1,000 V/cm voltage-to-distance ratio), 100 μs, 90 pulses as the clinically IRE protocols for comparison. As shown in Fig. 13, although the dose of 500 V pulses (2,250 V^2^s) was 51.54% larger than the dose (1,384.8 V^2^s) of combining SHV pulses and LLV (480 V) pulses, the ablation area (86.00 mm^2^) from combining SHV pulses and LLV (480 V) pulses was still 39.25% larger than that after 500 V pulses (61.76 mm^2^).

**Table 7:** Liver experimental parameters for the setups shown in Fig. 12, n = 3.

Parameters	SHV pulses			Lag time	LLV pulses			Sequence	Dose (V^2^s)	Ablation area (mm^2^)
	Voltage (V)	Width (μs)	Number		Voltage (V)	Width (μs)	Number			
Fig. 12					240	100	80	LLV	460.8	14.20 ± 4.72
	1,600	2	20	1 s	240	100	60	SHV + LLV	448	42.47 ± 9.74
					360	100	80	LLV	1,036.8	28.82 ± 6.45
	1,600	2	20	1 s	360	100	60	SHV + LLV	880	50.70 ± 1.34
					480	100	80	LLV	1,843.2	60.45 ± 10.63
	1,600	2	20	1 s	480	100	60	SHV + LLV	1,484.8	86.00 ± 8.51

**Table 8:** Liver ablation parameters for the setups shown in Fig. 13, n = 3.

Parameters	SHV pulses			Lag time	LLV pulses			Sequence	Dose (V^2^s)	Ablation area (mm^2^)
	Voltage (V)	Width (μs)	Number		Voltage (V)	Width (μs)	Number			
Fig. 13					500	100	90	LLV	2,250	61.76 ± 6.48
	1,600	2	20	1 s	480	100	60	SHV + LLV	1,484.8	86.00 ± 8.51

**Figure 14:**
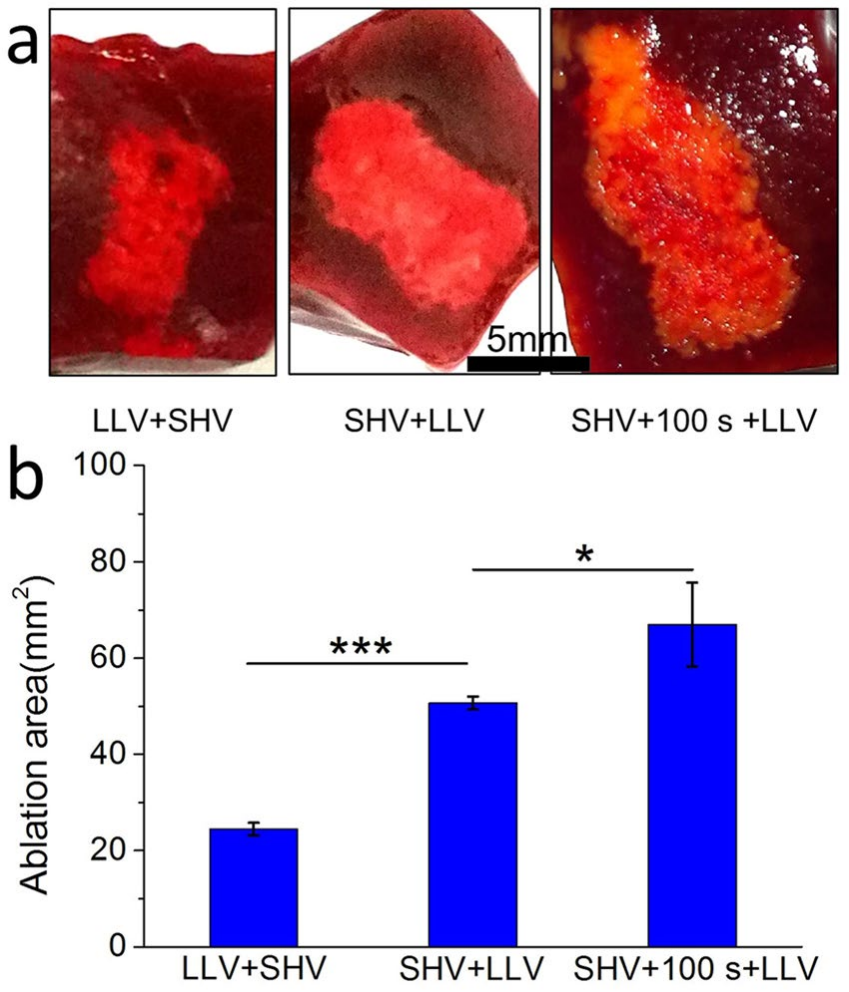
LLV + SHV pulses do not ablate rabbit liver as effectively as SHV + LLV pulses. Prolonging the lag time (100 s) also enlarged the ablation area (the parameters were given in Table 9). (**a**) Ablation areas of fresh liver samples and (**b**) Mean ablation areas induced from LLV + SHV pulses, SHV + LLV pulses, and SHV pulses + 100 s + LLV pulses. *p < 0.05, ***p < 0.001.

Pulse sequence plays an important role in cell cytotoxicity because of the synergistic effect of combining SHV pulses with LLV pulses. Therefore, the influence of the sequence on the liver ablation was also studied. As shown in Fig. 14, SHV + LLV (360 V) pulses induced an ablation area of 50.7 mm^2^. However, LLV (360 V) + SHV pulses created a 24.48 mm^2^ ablation area. There was also a significant difference (p < 0.001) between the different sequences of pulse protocols. Only SHV + LLV (360 V) pulses significantly enhanced the ablation area. The ablation area was further increased (67.03 mm^2^) by 32.21% when the lag time between SHV pulses and LLV (360 V) pulses was increased to 100 s compared to a lag time of 1 s. There was also a significant difference in ablation area (p < 0.05) between the sequences applied with different lag times (1 s and 100 s).

**Table 9:** Liver experimental parameters for the setups given in Fig. 14, n = 3.

Parameters	SHV pulses			Lag time	LLV pulses			Sequence	Ablation area (mm^2^)
	Voltage (V)	Width (μs)	Number		Voltage (V)	Width (μs)	Number		
Fig. 14	1,600	2	20	1 s	240	100	60	LLV + SHV	24.48 ± 1.29
	1,600	2	20	1 s	360	100	60	SHV + LLV	50.70 ± 1.34
	1,600	2	20	100 s	360	100	60	SHV + LLV	67.03 ± 8.73

**Figure 15:**
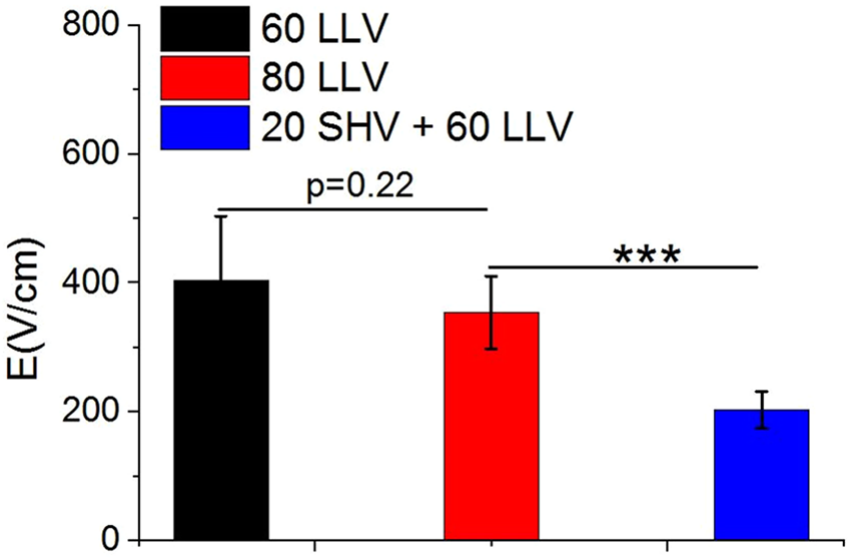
Lethal electric field thresholds for liver ablation induced by 60 LLV pulses and 80 LLV pulses were higher than that induced by 20 SHV pulses + 60 LLV pulses. ***p < 0.001, n = 9.

The lethal electric field thresholds are important to pulse selection in clinical applications. Combining SHV and LLV pulses had a lower lethal electric field threshold. As shown in Fig. 15, when the 60 LLV (~100 μs) pulses were applied, the lethal electric field threshold was 403 ± 100 V/cm. When the 80 LLV (~100 μs) pulses were applied, the lethal electric field threshold was 354 ± 56 V/cm. There was no statistically significant difference (p = 0.22) between the 60 and 80 LLV pulses on lethal electric field threshold. However, the lethal electric field threshold induced by combining 20 SHV pulses and 60 LLV pulses was 203 ± 28 V/cm, which was smaller than the lethal electric field threshold induced from 80 LLV (~100 μs) pulses. Moreover, there was also a significant statistical difference (p < 0.001) between 80 LLV pulses and 20 SHV + 60 LLV pulses.

**Figure 16:**
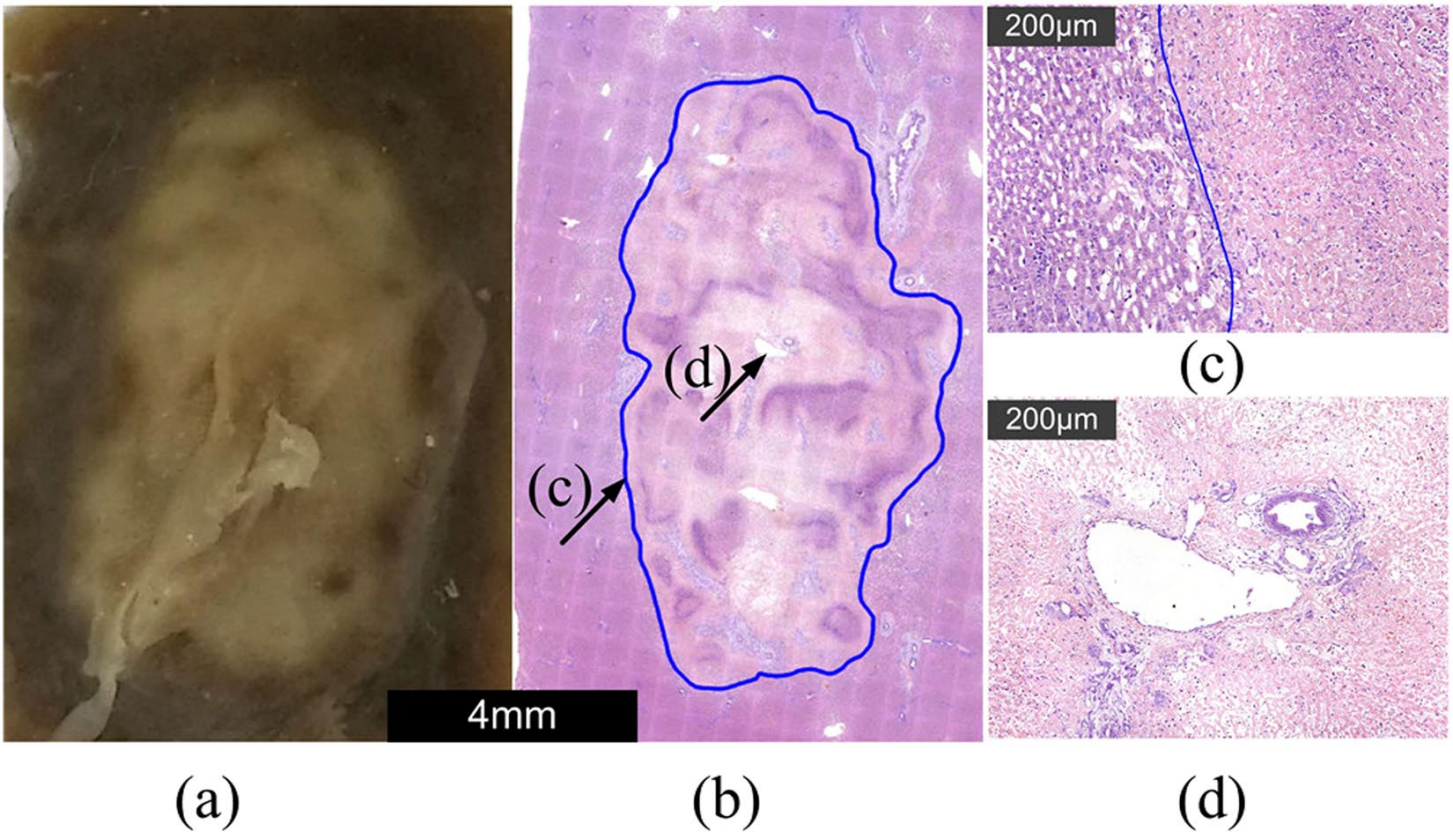
Tissue sections after (**a**) a fixation in 10% formalin and (**b**) hematoxylin and eosin staining (H&E) for the combination of SHV pulses with LLV (480 V) pulses. (**c**) The boundary between the ablation and normal tissue regions. (**d**) Tissue near a blood vessel and bile duct.

After a fixation in 10% formalin (Fig. 16(a)) and H&E staining and imaging (Fig. 16(b)), the boundaries between the ablation area and normal tissue regions after treatment with combined SHV pulses with LLV (480 V) pulses could be clearly and accurately observed. In addition, Fig. 16(c) showed an obvious boundary (μm-level) in the ablation region of the tissue. There was a clear demarcation between normal hepatocytes on the left compared to ablated cells on the right (solid line). Complete cell structures could not be observed in the ablation area, indicating that all the cells were necrotic, as shown in Fig. 16(c,d). However, blood vessels and bile ducts could still be seen in the ablation region. In particular, the structures of the blood vessels and bile duct were also complete. This outcome indicated that the ablation from SHV + LLV (480 V) pulses is also selective and does not affect blood vessels and bile ducts.

## Discussion

The mechanisms by which cell death occurs upon application of electric pulses continue to be actively investigated. One possible mechanism with experimental support posited lethal membrane disruption from repetitive electrical pulses^24^. The total electroporated area of the cell membrane that exceeds the critical transmembrane voltage is given by the following the equation^46^:

1 $${S}_{c}=S(1-\frac{2{U}_{c}}{3rE(1-{e}^{-t/\tau })})$$

where *S* is the total surface area of the cell membrane, *S*_*c*_ is the total electroporated area of the cell membrane, *E* is the external electric field, and *U*_*c*_ is the critical transmembrane voltage.

From equation (1), the total electroporated area of the cell membrane becomes larger with an increasing external electric field. Therefore, SHV pulses will induce a larger electroporated area in the cell membrane than LLV pulses. However, Pakhomov *et al*.^47^ showed that sub-microsecond pulses did not increase the size of pores formed in the membrane. Sano *et al*.^48^ reported that the delivery of ~1 μs pulses leads to the formation of numerous small pores rather than rapid cell membrane destruction caused by pore expansion. Moreover, as we calculated earlier^41^, short high-voltage also suggested that SHV (2 μs) pulses contribute less to pore development and produce small pores, which can recover easily after the pulses. In contrast, LLV pulses contribute more to pore development and create large pores. Neu *et al*.^49^ observed that low-voltage, long pulses create larger pores. In addition, Saulis *et al*.^50^ also found that a pulse of short duration creates smaller pores than a pulse of longer duration. However, because of the limitation of the critical transmembrane voltage, LLV pulses generate small electroporated areas, which limits further destruction of the cell membrane (Equation 1). Here, the combination of SHV pulses with LLV pulses takes advantage of both these processes; the SHV pulses create stronger electric field that induces a larger electroporated area in the cell membrane to reduce the limitation of the critical transmembrane voltage for LLV pulses; LLV pulses then create large pores at the electroporated region to further destroy the cell membrane, resulting in highly efficient cell ablation. As shown in Figs 3–5, cell cytotoxicity was significantly enhanced by combining SHV pulses with LLV pulses, which supports this hypothesis. It is worth noting that the sequence of SHV pulses and LLV pulses plays an important role: cell viability after application of SHV + LLV pulses is lower than when the pulse order is reversed, and significant differences were also found between different pulse sequences (Fig. 5). When LLV pulses were applied before SHV pulses, even if a large electroporated area in the cell membrane was created by the SHV pulses, there were no subsequent LLV pulses to contribute to pore expansion. Thus, LLV + SHV pulses induced cell death less effectively than SHV + LLV pulses.

Pakhomov *et al*.^51^ showed that delayed electrosensitization may occur after the first pulse train that renders cells more sensitive to the cytotoxic effect of the second pulse train when applied within a certain time interval, which increases cell death. Notably, the combination of SHV pulses with LLV pulses also evoked delayed electrosensitization for cell death. As shown in Fig. 3, when the lag time between SHV pulses and LLV pulses was increased to 100 s, the cell viability was further decreased compared to the pulse protocols with a 1-s lag time.

The combining SHV pulses with LLV pulses not only enhanced cell death but also significantly enlarged the tissue ablation area. The typical IRE treatment protocol uses ~100 μs pulses, and the ablation area is proportional to the pulse voltage. In this study, the LLV pulses followed traditional IRE protocols (~100 μs pulses). As shown in Fig. 7, the ablation area was expanded with an increased voltage of LLV pulses applied alone. When the electric pulses were applied to the pair of needle electrodes, high electric fields formed near the electrodes and tapered at distances from the electrodes. In general, the area is only affected by irreversible electroporation generating high electric fields near the electrode because of the critical electric field, and thus, the ablation area is restricted. However, adding short high-voltage (SHV) pulses before LLV pulses generates a large electroporated area because of a higher electric field on the liver tissue, which may reduce the effect on the limitation of the critical electric field for LLV pulses and thus extend the area of tissue ablation. As shown in Fig. 7, the synergistic effect of the combination of SHV pulses with LLV pulses generated a larger ablation area compared to SHV pulses or LLV pulses applied alone. Furthermore, as shown in Fig. 10, the sequence of the SHV and LLV pulses were important, and only SHV + LLV pulse treatments enhanced the ablation area. Even if an electroporated area of liver tissue was created by SHV pulses, there were no subsequent LLV pulses to ablate the tissue; therefore, the LLV + SHV pulse sequence enlarged the ablation area less effectively than the SHV + LLV pulse sequence. Muratori *et al*.^37^ found that evoked delayed electrosensitization by nanosecond pulses enhanced the ablation area in a 3D *in vitro* model, which suggested that delayed electrosensitization should occur *in vivo* as well. Subsequently, Muratori *et al*.^52^ found that electrosensitization occurs *in vivo* and can be exploited to assist in *in-vivo* cancer ablation. In this study, combining SHV pulses with LLV pulses also evoked delayed electrosensitization that further enlarged the liver ablation area in IRE therapy (Fig. 10), which indicated that delayed electrosensitization may also occur *in vivo*. However, the mechanism of delayed electrosensitization for enlargement of the ablation area by combining SHV pulses with LLV pulses is still being explored.

Rubinsky *et al*.^13^ considered the electric field threshold for IRE to be approximately 600 V/cm. Sano *et al*.^53^ found that the IRE electric field threshold in liver ablation was 423 V/cm. Miklavcic *et al*.^54^ also found that the IRE critical electric field was approximately 637 V/cm. In this study, the IRE electric field threshold was 354 ± 56 V/cm, which was in the range of 300 V/cm-500 V/cm according to the research of Edd *et al*.^55^. However, when combining SHV pulses and LLV pulses were applied, the lethal electric field threshold was 203 ± 28 V/cm, which was smaller than that of IRE pulses.

Guo *et al*.^56^ found that combining nanosecond pulses with millisecond pulses enhanced the efficiency of gene electrotransfer. Interestingly, they also found that cell viability was markedly decreased by this combination compared to millisecond pulses applied alone. Zgalin *et al*.^57^ found that combining nanosecond pulses with microsecond pulses increased the level of inactivation of Escherichia coli in water samples compared to nanosecond pulses or microsecond pulses applied alone. In this study, we also found that combining short microsecond (2 μs) pulses with long microsecond (100 μs) pulses enhanced cell cytotoxicity and increased the ablation area compared to short microsecond (2 μs) pulses or long microsecond (100 μs) pulses applied alone. Nanosecond electric pulses impair the barrier function of the cell membrane and endoplasmic reticulum and mitochondrial membranes, which induce necrosis and apoptosis. However, Gowrishankar *et al*.^58^ found that nanosecond pulses generate larger electroporated areas in the cell membrane due to the higher electric field. We hypothesized in this study that nanosecond pulses may induce a stronger electric field and thus create a larger electroporated area, making the electroporated area more susceptible to LLV pulses and ultimately resulting in highly efficient necrosis. Therefore, combining nanosecond pulses with microsecond pulses could be further investigated to promote the synergistic effect to enhance cell cytotoxicity and enlarge the tissue ablation area.

In this study, we show that an optimized combination of SHV pulses and LLV pulses may provide therapeutic benefits from IRE protocols and enhance the outcome of IRE efficacies. However, the results of this *in vitro* or *in vivo* pilot study warrant further exploration regarding the combination of SHV pulses with LLV pulses for use as a clinical tool. Further pulse parameter optimization is also needed to maximize the ablation area. Studies in large animals that use clinical needle electrodes with more than a 1 cm distance and a higher voltage pulse generator should also be conducted to determine the maximum ablation sizes that are achievable by using the combination of SHV pulses with LLV pulses. This study also suggests a possible mechanism for the observed synergistic effects. Therefore, further experimental validation of the hypothesis regarding the synergistic effects at the single-cell level should be performed.

## Conclusion

This study showed that a synergistic effect was evoked by a combination of SHV pulses with LLV pulses that enhanced cell cytotoxicity and enlarged the ablation area. The results showed that combining SHV pulses with LLV pulses significantly decreased cell viability compared to SHV pulses or LLV pulses applied alone. The combination of SHV pulses with LLV pulses also created a larger ablation area in the livers of rabbits than SHV pulses or LLV pulses applied alone. Cell cytotoxicity and the ablation area were further enhanced because of delayed electrosensitization. However, the sequence of SHV and LLV pulses mattered, and the application of LLV + SHV pulses did not decrease cell viability or increase the ablation area. A hypothesis was also suggested regarding the synergistic effect. This study provides evidence that a combination of SHV pulses with LLV pulses can be applied to optimize current clinical IRE protocols to enhance IRE efficacy, and further investigations are warranted.

## Electronic supplementary material

Supplementary Information
