# Supplementary material for: Synergistic combinations of short high-voltage pulses and long low-voltage pulses enhance irreversible electroporation efficacy
Source: Sci Rep. 2017 Nov 9;7:15123. doi: 10.1038/s41598-017-15494-3 (PMC5680269; doi:10.1038/s41598-017-15494-3)
Supplement: Supplementary file 1 — Supplementary Information [file 41598_2017_15494_MOESM1_ESM.pdf]

Synergistic combinations of short high-voltage pulses and long low-voltage pulses enhance irreversible electroporation efficacy

Chenguo Yao[1*], Yanpeng Lv[1*], Yajun Zhao[1], Shoulong Dong[1], Hongmei Liu[1], Jianhao Ma[1]

[1]the State Key Laboratory of Power Transmission Equipment and System Security and New Technology, the School of Electrical Engineering, Chongqing University, Chongqing 400030, China, *These authors contributed equally to this work. Correspondence and requests for materials should be addressed to Chenguo Yao (email: yaochenguo@cqu.edu.cn) or Yanpeng Lv (email: lvyanpeng@cqu.edu.cn)

Materials and Methods

Analysis of Electric Field threshold and temperature distribution in rabbit liver

Finite element models were created in COMSOL Multiphysics to solve the electric field and temperature distribution in the liver tissue region[1, 2]. The liver tissue was modeled as a 20 mm length square, the liver conductivity and dielectric constant were set up as constant to simplify the simulation[3]. A pair of 1 mm diameter electrodes were inserted into tissues and their edge-to-edge distance was equal to 4 mm (Supplemental Figure S1). Within the solution domain, the Electric Currents module was used to solve for following equations:

$$\nabla \cdot J = Q_j \left[\frac{A}{m^3}\right] \quad (1)$$

$$J = \left(\sigma + \varepsilon_0 \varepsilon_r \frac{\partial}{\partial t}\right) E \left[\frac{A}{m^2}\right] \quad (2)$$

$$E = -\nabla U \left[\frac{V}{m}\right] \quad (3)$$

where U is the electric potential, E is the electric field, J is the current density, Q is the current source, σ is the conductivity, ε_r is the relative permittivity, and ε_0 is the permittivity of free space. The boundaries surrounding one electrode were assigned a constant electrical potential

$$U = U[V] \quad (4)$$

The boundaries of the other electrode were assigned as a relative ground

$$U = 0[V] \quad (5)$$

The remaining boundaries were defined as electrical insulation

$$n \cdot J = 0\left[\frac{A}{m}\right] \quad (6)$$

where n is the normal vector to the surface.

Changes in temperature due to Joule heating were calculated for combining SHV pulses and LLV pulses using a modified duty cycle approach[3-5]. The temperature distribution (T) was obtained by transiently solving a heat conduction equation:

$$\rho c \frac{\partial \boldsymbol{T}}{\partial t} = \nabla \cdot (\mathrm{k} \nabla \boldsymbol{T}) + \frac{\tau(\sigma |\boldsymbol{E}|^2)}{p} [\frac{J}{m^3 \cdot s}] \quad (7)$$

where τ is the pulse duration, p is the period of the pulses, k is the thermal conductivity, c is the specific heat at constant pressure, and ρ is the density. Outer boundaries were treated as convective cooling:

$$-n \cdot (-\mathrm{k} \nabla \boldsymbol{T}) = h(\mathrm{T}_{ext} - T)[\frac{W}{m^2}] \quad (8)$$

with an exterior temperature (T_{ext}) of 38 ℃ and a heat transfer coefficient (h) of 25 (W m^{-2} K^{-1}). Parameter values for these simulations can be found in Supplemental Table S1. The distribution of electric field was shown in Supplemental Figure S2

Supplemental Table S1: Parameters used in finite element models

Definition	Symbol	Rabbit liver	Reference	Electrode	Reference	Unit
**Conductivity**	σ	0.067	6	4.03×10^{6}	3	[S/m]
**Relative permittivity**	ε_r	42672	6	1	3	
**Thermal conductivity**	k	0.52	5	44.5	3	[W/(m·k)]
**Specific heat capacity**	c	3540	5	475	3	[J/(kg/k)]
**Density**	ρ	1079	5	7850	3	[kg/m^3]

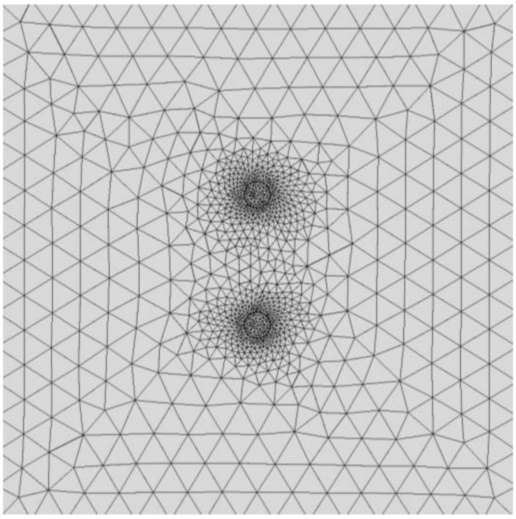

Supplemental Figure S1: Finite element mesh used to calculate the electric field and temperature distribution within a simulated tissue domain.

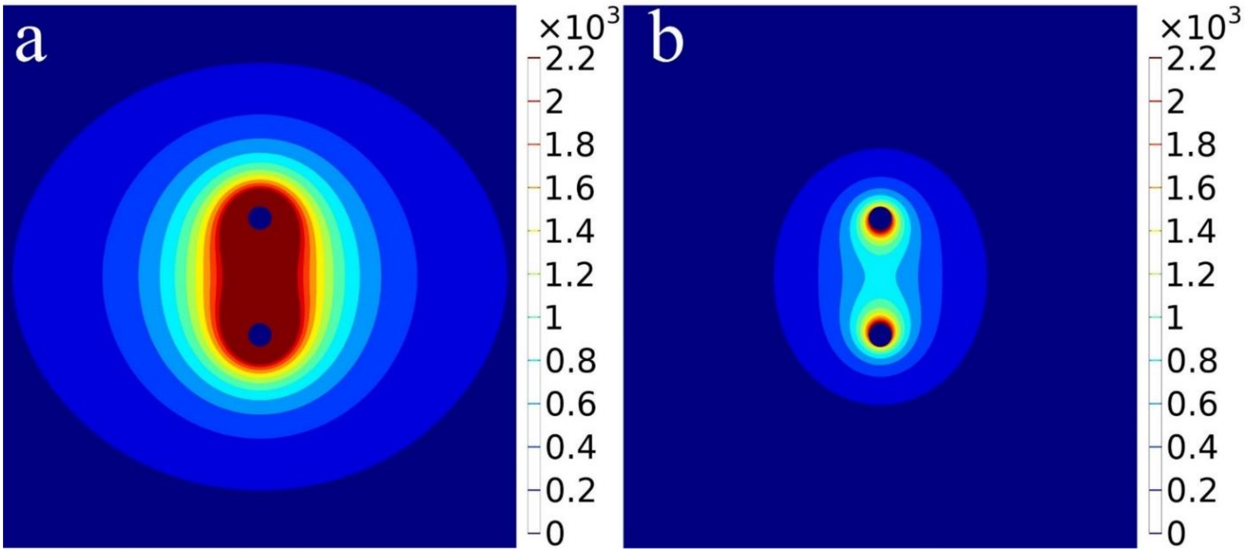

Supplemental Figure S2: The distribution of electric field (V/cm) for 1600 V (a) and 480 V (b) applied.

References

1. Muratori, C. et al. Electrosensitization Increases Antitumor Effectiveness of Nanosecond Pulsed Electric Fields in Vivo. Technol Cancer Res Treat. 1875980045 (2017).
2. Nuccitelli, R. et al. Nanoelectroablation of Murine Tumors Triggers a CD8-Dependent Inhibition of Secondary Tumor Growth. PLOS ONE. 10, e134364 (2015).
3. Sano, M. B. et al. Bursts of Bipolar Microsecond Pulses Inhibit Tumor Growth. Scientific reports 5 (2015).
4. Arena, C. B. et al. High-frequency irreversible electroporation (H-FIRE) for non-thermal ablation without muscle contraction. Biomed Eng Online 10, 102 (2011).
5. Neal, R. E., 2nd, Garcia, P. A., Robertson, J. L. & Davalos, R. V. Experimental Characterization and Numerical Modeling of Tissue Electrical Conductivity during Pulsed Electric Fields for Irreversible Electroporation Treatment Planning. IEEE Trans Biomed Eng 59, 1076–1085 (2012).
6. Sel, D. et al. Sequential Finite Element Model of Tissue Electropermeabilization. IEEE Trans Biomed Eng. 52, 816-827 (2005).
